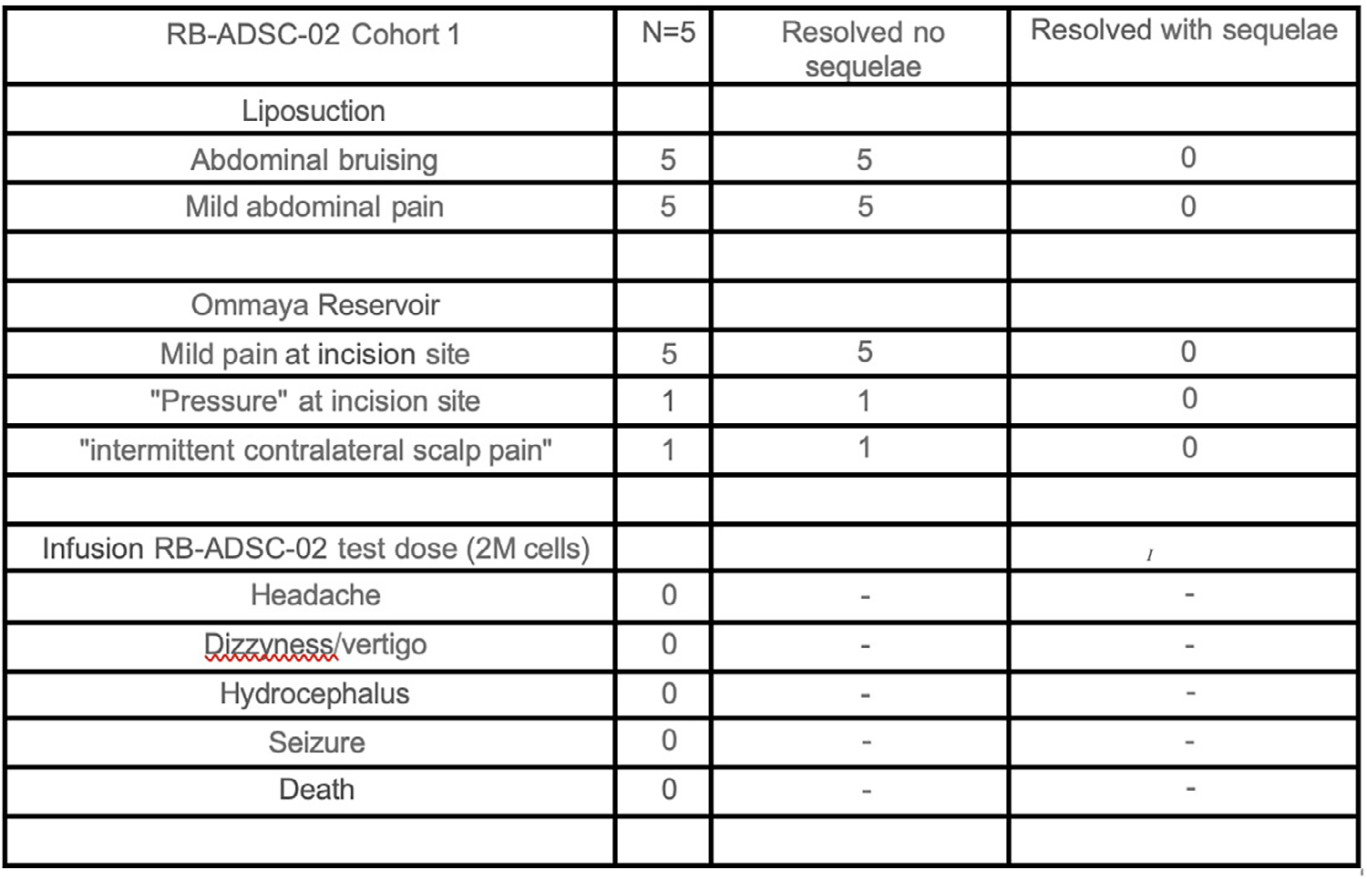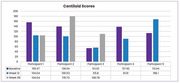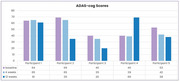# Early results of a "First‐in‐Human" Phase 1 clinical study of intracerebroventricular injections of ex vivo expanded, autologous, Wnt‐activated, adipose‐derived stem cells in participants with mild to moderate Alzheimer's Disease (AD)

**DOI:** 10.1002/alz70861_108493

**Published:** 2025-12-23

**Authors:** Christopher M. Duma, Hans Keirstead, Gabriel Nistor, Robert Lynn, Jessica J. Buxton, Zoe M Hareng, Karlyssa Chung, Gustavo Alva, Sawyer H. Farmer, Ashley Harris

**Affiliations:** ^1^ Regeneration Biomedical, Inc., Newport Beach, CA USA; ^2^ University of California, Berkeley, Berkeley, CA USA; ^3^ Regeneration Biomedical, Inc., NEWPORT BEACH, CA USA; ^4^ ATP Clinical Research, Costa Mesa, CA USA; ^5^ Albany Medical College, Albany, NY USA; ^6^ Brain and Spine Surgeons of Orange County, Newport Beach, CA USA

## Abstract

**Background:**

There is very little reported human experience with cell therapy for AD. We test the safety of using Wnt‐activated adipose tissue‐derived stem cells (ADSCs) injected directly into the ventricles of the brain to address multifactorial etiologies.

**Method:**

Two million and 5 million expanded, Wnt‐expressing, ADSCs were tested in the first 5 patients ("low‐dose and medium‐dose") of a Phase 1 FDA clinical trial using escalating doses. Participants had age <80 years, FAST stages 4 and 5, and cognitive, amyloid PET centiloid scores, and CSF analyses consistent with AD. The participants underwent: 1) lipoaspiration and cell preparation, 2) Ommaya reservoir implantation, and 3) infusion of a *single* dose of the test product via Ommaya reservoir. The primary endpoint was safety. Secondary endpoints included normalization of phosphorylated tau (*p* ‐Tau), and amyloid beta, and improvement in cognitive testing scores.

**Result:**

Adverse events (AEs) from pre‐injection surgical procedures included mild bruising and discomfort. Test product injection showed no AEs (range: 23‐55 weeks) including headache or nausea (Figure 1). At 12 weeks post‐injection, CSF phosphorylated tau (*p* ‐Tau) improved in 80% of the participants from a median of 60.2 pg/ml (range: 46.9 ‐ 76.1) to a normal median of 36.8 (range 15.0 – 66.6), amyloid PET scan centiloid scores decreased in 60% of participants from a pre‐injection median of 137.2 (range: 55.33 – 155.47) to a median of 100.53 (range: 55.58 – 168.1, Figure 3). ADAS‐cog scores improved in 80% of participants by week 12 from a pre‐injection median of 53 (range: 40 – 69) to a median of 38 (range: 20 – 69, Figure 4), MMSE scores improved in 60% of participants by week 12 from a pre‐injection median of 16 (range: 14 – 19) to a median of 18 (range: 12 – 20).

**Conclusion:**

This "first‐in‐human" trial of intracerebroventricular injection of expanded autologous, Wnt‐expressing, adipose tissue‐derived stem cells proved well‐tolerated and safe for the low‐dose and medium dose cohorts. Secondary endpoints showed promising improvements.